# Comparing efficacy and safety of P013, a proposed pertuzumab biosimilar, with the reference product in HER2-positive breast cancer patients: a randomized, phase III, equivalency clinical trial

**DOI:** 10.1186/s12885-022-09895-5

**Published:** 2022-09-07

**Authors:** Abolghasem Allahyari, Ali Ehsanpour, Nafiseh Ansarinejad, Valiollah Mehrzad, Behjat Kalantari, Jahangir Raafat, Mojtaba Ghadiany, Farhad Shahi, Behrooz Gharib, Vahid Moazed, Adnan Khosravi, Mir Hossein Mirpour, Sina Salari, Seyedmohammadreza Mortazavizadeh, Amirabbas Nekoyi, Mohsen Khani, Alireza Sadeghi, Sirus Gharib, Alireza Bary, Mehrzad Mirzania, Shirin Haghighat, Seyed Mohsen Razavi, Seyed Amir Hossein Emami, Mehran Hosseinzadeh, Mahdi Mirbolouk, Sanambar Sadighi, Abdolali Shahrasbi, Ali Esfahani, Masoumeh Gity, Nassim Anjidani, Hamidreza Kafi, Safa Najafi

**Affiliations:** 1 Hematology Oncology Department, Faculty of Medicine, Mashhad University of Medical Sciences, Mashhad, Iran; 2 Thalassemia and Hemoglobinopathy Research Center, Health Research Institute, Ahvaz Jundishapur University of Medical Sciences, Ahvaz, Iran; 3 Department of Hematology & Oncology, Iran University of Medical Sciences, Tehran, Iran; 4 Hematology and Oncology Department, Isfahan Medical School, Isfahan, Iran; 5 Hematology & Oncology, Department of Internal Medicine, School of Medicine, Shahid Bahonar Hospital, Kerman University of Medical Sciences, Kerman, Iran; 6 Mehrad Hospital, Tehran, Iran; 7 Department of Hematology and Medical Oncology, Shahid Beheshti University of Medical Sciences, Tehran, Iran; 8 Department of Hematology and Medical Oncology, Breast Disease Research Center, Cancer Institute, Imam Khomeini Hospital Complex, Tehran University of Medical Sciences, TUMS, Tehran, Iran; 9 Department of Medical Oncology & Hematology, Naft Hospital, Tehran, Iran; 10 Hematology & Oncology Kerman University of Medical Sciences, Kerman, Iran; 11 Hematology & Oncology, Department of Adult Hematology & Oncology, School of Medicine, Chronic Respiratory Diseases Research Center, National Research Institute of Tuberculosis and Lung Diseases, Dr. Masih Daneshvari Hospital, Shahid Beheshti University of Medical Sciences, Tehran, Iran; 12 Medical Oncology Guilan University of Medical Sciences, Razi Hospital, Fuman, Iran; 13 Medical Oncology, Hematology Taleghani Hospital, Shahid Beheshti University of Medical Sciences, Tehran, Iran; 14 Hematology/Oncology Yazd Azad University, Yazd, Iran; 15 Department of Hematology and Medical Oncology, Seyyed-al-shohada Hospital, Esfahan University of Medical Sciences, Tehran, Iran; 16 Medicine Saba Oncology Clinic, Isfahan, Iran; 17 Department of Hematology-Oncology, Isfahan University of Medical Sciences, Isfahan, Iran; 18 Hematology, Oncology Department of Internal Medicine, Guilan University of Medical Sciences, Guilan, Iran; 19 Department of Hematology & Oncology, Ghaem Hospital, Mashhad University of Medical Sciences, Mashhad, Iran; 20 Department of Hematology and Medical Oncology, Cancer Research Center, Cancer Institute of Iran, Imam Khomeini Hospital Complex, Tehran University of Medical Sciences, Tehran, Iran; 21 Hematology Research Center, Hematology and Medical Oncology Department, Shiraz University of Medical Sciences, Shiraz, Iran; 22 Iran Medical Science University, Tehran, Iran; 23 Medical Oncology, Cancer Institute, Imam Khomeini Hospital, School of Medicine, Tehran University of Medical Sciences, Tehran, Iran; 24 Hematology, Oncology Department of Internal Medicine, School of Medicine, Ahvaz Jundishapur University of Medical Sciences, Ahvaz, Iran; 25 Hazrat Rasol Hospital Rasht, Gilan, Iran; 26 Internal Medicine Group TUMS Faculty of Cancer Institute of Iran, Tehran, Iran; 27 Bouali Hospital, Tehran Medical Sciences, Islamic Azad University, Tehran, Iran; 28 Hematology and Oncology Research Center, Tabriz University of Medical Sciences, Tabriz, Iran; 29 Advanced Diagnostic and Interventional Radiology Research Center, Breast Disease Research Center, Imam Khomeini Hospital Complex, Tehran University of Medical Sciences, Tehran, Iran; 30 Medical Department, Orchid Pharmed Company, Tehran, Iran; 31 Breast Cancer Research Center, Motamed Cancer Institute, ACECR, Tehran, Iran

**Keywords:** Breast Cancer, Pertuzumab, Biosimilar, Equivalency, Randomized clinical trial

## Abstract

**Background:**

Breast cancer is the most frequently diagnosed cancer and the leading reason for cancer-related death among women. Neoadjuvant treatment with dual-HER2 (human epidermal growth factor receptor 2) blockade has shown promising effects in this regard. The present study aimed to compare the efficacy and safety of a proposed pertuzumab biosimilar with the reference pertuzumab.

**Methods:**

This randomized, phase III, multicenter, equivalency clinical trial was conducted on chemotherapy-naive women with HER2-positive breast cancer. Patients were randomly assigned (1:1) to receive six cycles of either P013 (CinnaGen, Iran) or the originator product (Perjeta, Roche, Switzerland) along with trastuzumab, carboplatin, and docetaxel every 3 weeks. Patients were stratified by cancer type (operable, locally advanced, inflammatory) and hormone receptor status. The primary endpoint was breast pathologic complete response (bpCR). Secondary endpoints included comparisons of total pCR, overall response rate (ORR), breast-conserving surgery (BCS), safety, and immunogenicity.

**Results:**

Two hundred fourteen patients were randomized to treatment groups. bpCR rate in the per-protocol population was 67.62% in the P013 and 71.57% in the reference drug groups. Based on bpCR, P013 was equivalent to the reference pertuzumab with a mean difference of − 0.04 (95% CI: − 0.16, 0.09). Secondary endpoints were also comparable between the two groups.

**Conclusions:**

The proposed biosimilar P013 was equivalent to the reference product in terms of efficacy. The safety of both medications was also comparable.

**Supplementary Information:**

The online version contains supplementary material available at 10.1186/s12885-022-09895-5.

## Background

Breast cancer is the most frequently diagnosed cancer and the leading reason for cancer-related death among women [1]. HER2 is amplified in 15–25% of breast cancers [2, 3], resulting in an aggressive disease [4]. Hence, HER2-positive breast cancer patients will benefit from adding HER2-targeted therapies to their chemotherapy regimen [5]. Neoadjuvant treatment results in downstaging the tumor, reducing the risk of distant recurrence and facilitating surgery; therefore, patients may undergo breast-conserving surgery instead of mastectomy [6, 7].

Pathological complete response (pCR) is a useful assessment to evaluate the efficacy of newly developed drugs administered in neoadjuvant settings and is believed to be a surrogate endpoint for accelerated drug approval for early-stage breast cancer [8]. The acceptable pCR rate is associated with long-term efficacy [9, 10].

Trastuzumab is the first HER2-directed humanized monoclonal antibody approved by the FDA to be used against solid tumors. Trastuzumab administration in operable breast cancer contributes to improved disease-free survival (DFS), overall survival (OS) [11, 12], and pCR [13, 14]. Pertuzumab, another anti-HER2 therapy, binds to domain II of HER2 receptor and blocks ligand-dependent dimerization, resulting in complete inhibition of HER2 signaling [15]. Furthermore, the addition of pertuzumab to trastuzumab in neoadjuvant setting, in significant improvement in patients’ OS [13].

According to the FDA, a biosimilar is a biological product that is highly similar to an existing approved originator product, with no clinically significant differences with regards to safety, purity, and potency. Biosimilar manufacturing decreases care costs, provides easier access to lifesaving biological medications, that leads to availability of more therapeutic options [16, 17].

P013 (CinnaGen Company, Iran) is a proposed biosimilar to originator pertuzumab (Perjeta, Roche, Switzerland). Preclinical studies of P013 are performed in terms of general toxicity only. According to EMA guideline, pharmacokinetic assessments are not required in phase III studies [18].

The aim of this study was to compare P013 with reference pertuzumab in terms of efficacy, safety, and immunogenicity in early HER2-positive breast cancer patients being treated in the neoadjuvant setting.

## Methods

### Study design

This study was a phase III, multicenter, triple-blind, equivalency trial to compare the therapeutic efficacy and safety of P013 to originator pertuzumab in early HER2-positive breast cancer patients.

The study was performed in accordance with Good Clinical Practice (GCP) guidelines and the Declaration of Helsinki. Each participant signed a written informed-consent form before the initiation of the trial. The Research Ethics Committee of Tehran University of Medical Sciences (IR.TUMS.VCR.REC.1397.127) and the Research Ethics Committee of Guilan University of Medical Sciences (IR.GUMS.REC.1395.444) approved the study protocol. The study has been registered in Clinicaltrials.gov (NCT04957212, first registered on 12/07/2021). This trial has also been registered in Iranian Registry of Clinical Trials (IRCT) with the number of IRCT20150303021315N11 (11/06/2018).

### Intervention

The study drugs were administered intravenously on a 3-weekly schedule and were given consecutively on the same day in the following order: trastuzumab, followed by pertuzumab, carboplatin, and docetaxel (TCHP regimen). Patients received trastuzumab at an initial dose of 8 mg/kg, followed by 6 mg/kg; pertuzumab at an initial dose of 840 mg, followed by 420 mg. Carboplatin was administered based on the area under the plasma-concentration time curve (AUC) 6, and docetaxel was given at 75 mg/m^2^. Treatment was continued for six cycles and then was followed by surgery.

### Patients

Female patients aged between 18 and 70 years with operable (T2–3, N0–1, M0), locally advanced (T2–3, N2–3, M0 or T4a-c, any N, M0), or inflammatory (T4d, any N, M0) breast cancer were eligible for the study. HER2 positivity was confirmed with Immunohistochemistry (IHC) 3+ or positive Fluorescence or Chromogenic in situ hybridizations (FISH/CISH) for IHC 2+ tumors. Further inclusion criteria were primary tumor size of more than 2 cm in diameter, Eastern Cooperative Oncology Group (ECOG) performance status of 0 or 1, and signed informed consent.

Key exclusion criteria were as follows: metastatic disease (stage IV) or bilateral breast cancer; The presence of other malignancies (except for carcinoma in situ of the cervix or basal cell carcinoma); previous anticancer therapy or radiotherapy for any malignancy, history of major surgery within 4 weeks of randomization; organ dysfunction; pregnancy, lactation, and refusal to use contraception.

Other exclusion criteria were uncontrolled hypertension, unstable angina, congestive heart failure (CHF), severe cardiac arrhythmia requiring treatment, history of myocardial infarction within 6 months of enrollment; any other severe uncontrolled systemic disease; dyspnea at rest or other illnesses which required continuous oxygen therapy.

### Randomization and masking

Patients were stratified dynamically according to two factors: type of breast cancer and estrogen/ progesterone receptor (ER/PR) with 1:1 allocation ratio.

Patients were randomly assigned to treatment by a central randomization procedure for each consecutive eligible patient. We Allocated randomization codes after signing of the informed consent form, approval of all eligibility criteria and identification of stratification factors.

### Outcomes

The primary endpoint was breast pCR (bpCR), defined as the absence of invasive neoplastic cells at the microscopic examination of the primary tumor at surgery following prior systemic therapy (ypT0/is). All the centers performed similar surgery procedures; moreover, the methodology used for pCR assessment was the same among pathologists. Each center had only one surgeon and pathologist.

Secondary efficacy endpoints evaluable at the end of the neoadjuvant period included total pCR (tpCR), defined as no invasive tumor residues in the breast and lymph nodes (ypT0/is ypN0); objective response rate (ORR) and rate of breast-conserving surgery (BCS) for patients whom mastectomy was planned before treatment (T2–3). ORR, defined as clinical Complete Response (cCR) or clinical Partial Response (cPR) by Response Evaluation Criteria in Solid Tumours version 1.1 (RECIST 1.1) [19], was assessed by a blinded central radiologist with the comparison of breast magnetic resonance imaging (MRI) before and after completion of neoadjuvant treatment.

During this study, adverse events (AEs) were monitored continuously, and all the reported events were graded according to the National Cancer Institute Common Terminology Criteria for Adverse Events (CTCAE) v5.0. The causality relation was assessed based on World Health Organization (WHO) criteria.

In this study, a decrease in left ventricular ejection fraction (LVEF) was considered as the only adverse event of special interest (AESI); therefore, LVEF was monitored and assessed by echocardiography in particular intervals throughout the study. LVEF measurements were conducted at baseline and every 6 weeks during the treatment.

For immunogenicity assessment, blood samples were collected before all treatment cycles and 3 weeks after the last administration. The enzyme-linked immunosorbent assay (ELISA) method was used for anti-drug antibodies (ADAs) measurement.

### Statistical analysis

A sample size of 107 in each group was assumed to provide at least 80% power to detect equivalency when assessed by risk difference between groups for a pCR with a predefined margin of 0.20, that 66% of patients would achieve a pCR in the reference group and 10% drop-out rate [20].

The intention-to-treat (ITT) population was defined as all patients who were randomly allocated to study groups, regardless of receiving the whole doses or not. The per-protocol (PP) population comprised all patients in the ITT population, except for those who had a major protocol deviation or did not receive at least three doses of study medication.

The primary outcome was first evaluated in the PP population and then in the ITT population as a sensitivity analysis. In imputation for pCR, patients with no available data were considered as non-responders.

A two-sided 95% confidence interval (CI) for the difference of pCRs was calculated based on the proportion test with no adjustment for covariates. The equivalency between the test product and reference product was claimed if the 95% CI of the difference of pCR proportions was completely within the pre-determined acceptance limits of − 0.2 and 0.2. For the secondary efficacy analysis, descriptive statistics were used. Moreover, the secondary efficacy outcomes in two treatment groups were compared using the chi-square or Fisher’s exact test.

Safety, demographic, and other characteristics data were summarized using descriptive statistics. The safety set was defined as all patients who received at least one dose of the study treatment during the study. Statistical analysis was performed using STATA software (version 14, StataCorp LP, USA) and Rstudio software (RStudio Inc., USA).

## Results

### Patient disposition and baseline characteristics

**Fig. 1:**
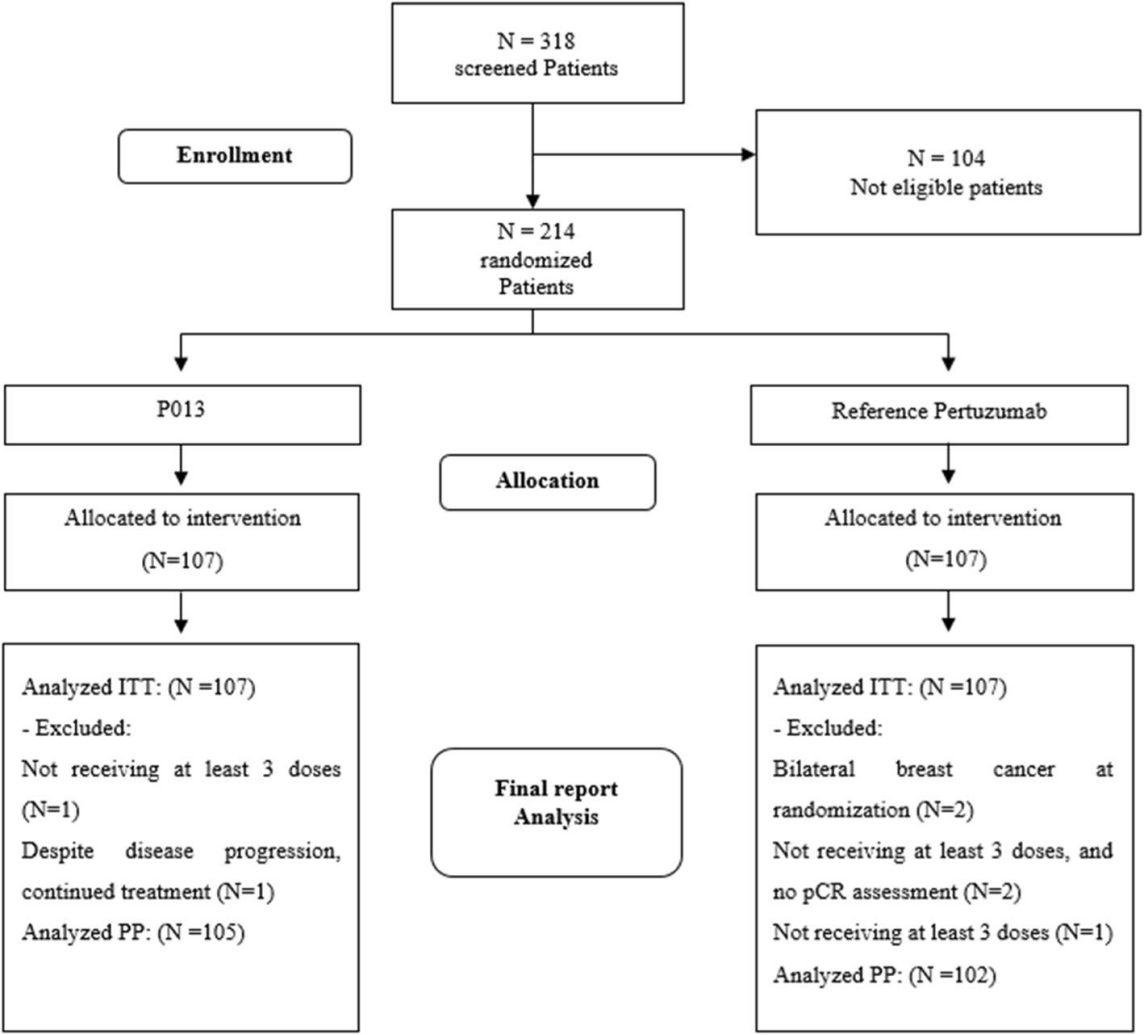
Patient disposition

Of the 318 patients screened, 214 patients were randomized to treatment groups in a 1:1 allocation ratio between August 2018 and May 2020 across 41 centers in 9 cities. A total of 206 (96.26%) patients received all six chemotherapy cycles and underwent surgery. The patient disposition scheme is demonstrated in Fig. 1.

Demographics and baseline characteristics of the patients were matched between the two groups (Table 1).

**Table 1:** Demographics and baseline characteristics of the participants

	P013 (***N*** = 107)	Reference pertuzumab (***N*** = 107)
**Age (years),** Mean ± SD	47.56 ± 10.31	44.35 ± 9.81
**BMI (kg/m**^**2**^**),** Mean ± SD	28.27 ± 5.30	27.27 ± 4.38
**BSA (m**^**2**^**),** Mean ± SD	1.79 ± 0.18	1.79 ± 0.18
**IHC 2+,** No. (%)		
FISH+	3 (2.80)	3 (2.80)
CISH+	6 (5.61)	3 (2.80)
**IHC 3+,** No. (%)	98 (91.59)	101 (94.39)
**ER/PR,** No. (%)		
ER and/or PR positive	63 (58.88)	64 (59.81)
ER and PR negative	44 (41.12)	43 (40.19)
**Type of breast cancer,** No. (%)		
Inflammatory	6 (5.61)	6 (5.61)
Locally advanced	49 (45.79)	48 (44.86)
Operable	52 (48.60)	53 (49.53)
**The largest dimension of the tumor size, median (Q1, Q3)**	45 (34, 65)	45 (35, 63)

There is no imputation for missing values

*BMI* Body Mass Index, *BSA* Body Surface Area, *IHC* Immunohistochemistry, *FISH* Fluorescence in situ hybridization, *CISH* Chromogenic in situ hybridization, *ER/PR* Eestrogen/ Progesterone receptor

### Efficacy

#### Pathological complete response

bpCR was analyzed as the primary outcome in ITT and PP populations (Table 2). bpCR rate in the PP population was 67.62% (71 patients) in P013 and 71.57% (73 patients) in the reference pertuzumab group. Therefore, with a mean difference (95% CI) of − 0.04 (− 0.16, 0.09) for difference, P013 is equivalent to the reference pertuzumab.

**Table 2:** bpCR analyses in the PP and ITT populations

	P013	Reference pertuzumab	Difference (95% Confidence interval)
Breast pCR (PP)	71 (67.62)	73 (71.57)	−0.04 (− 0.16, 0.09)
Breast pCR (ITT)	71 (66.36)	74 (69.16)	−0.03 (− 0.15, 0.10)
Breast pCR according to ER/PR (ITT)			
ER/PR + (*N* = 127)	35 (55.55)	38 (59.37)	−0.04 (− 0.21, 0.13)
ER/PR - (*N* = 87)	36 (81.82)	36 (83.72)	−0.02 (− 0.18, 0.14)

For ITT analysis, patients with missing assessments were considered to be nonresponders

Difference = P013- Reference pertuzumab

*pCR* Pathological complete response, *ER/PR* Eestrogen/ Progesterone receptor

As shown in Table 2, the difference in bpCR (P013-reference pertuzumab) was not significant in both HR-positive and -negative subgroups.

According to the prespecified margin of ±0.2, the equivalency of two medications was claimed as the 95% CI for the difference between the pCR proportions of the two groups, was entirely within the limits of − 0.2 and 0.2.

### Secondary outcomes

#### tpCR

tpCR was analyzed as the secondary outcome in ITT population. tpCR was reported in 56.07% (60 patients) in P013 and 63.55% (68 patients) in the reference pertuzumab group (mean difference (95% CI) = − 0.07 (− 0.21, 0.06)), as shown in Table 3. The difference between the two groups was not statistically significant (*P*-value = 0.26).

**Table 3:** pCR analyses in the ITT populations

	P013	Reference pertuzumab	Difference (95% Confidence interval)
Total pCR (ITT)	60 (56.07)	68 (63.55)	−0.07 (− 0.21, 0.06)

For ITT analysis, patients with missing assessments were considered to be nonresponders

Difference = P013- Reference pertuzumab

*pCR* Pathological complete response

#### ORR

ORR was 87.85% in the P013 arm, with 64 patients showing CR and 30 patients showing PR. The proportion of patients with ORR was 84.11% in the reference pertuzumab group (CR: 61, PR: 29 patients). All clinical responses among the two groups are presented in Table 4.

**Table 4:** Proportion of Clinical Response in both groups

	P013 (***N*** = 107)	Reference pertuzumab (***N*** = 107)
**Complete Response**	64 (59.81)	61 (57.01)
**Partial Response**	30 (28.04)	29 (27.10)
**Progressive Disease**	2 (1.87)	3 (2.80)
**Stable Disease**	3 (2.80)	2 (1.87)
**Unknown**	8 (7.48)	12 (11.21)

*P*-value = 0.99 (Fisher’s Exact test)

Data are presented as No. (%)

#### BCS

From 64 patients who were candidates for mastectomy in the P013 group, 20 patients (31.25%) underwent BCS, and 21 patients out of 59 mastectomy candidates (35.59%) in the reference pertuzumab group had lumpectomy. There was no statistically significant difference between the both groups regarding BCS rate (*p*-value = 0.56).

#### Safety

In this study, the total number of AEs was 2075 (50.56%) in the P013 arm, compared to 2029 (49.44%) in the reference arm. The most reported AEs were Anaemia, Thrombocytopenia, and Nausea in both arms. Table 5 demonstrates the ten most common reported AEs. Most of the AEs were of grades 1 or 2.

**Table 5:** Adverse event (Safety population)

Variable	P013 (***N*** = 107)	Reference pertuzumab (***N*** = 107)
**The most common AEs**		
Anaemia	103 (96.26)	100 (93.46)
Thrombocytopenia	69 (64.49)	70 (65.42)
Nausea	64 (59.81)	71 (66.36)
Diarrhoea	61 (57.01)	67 (62.62)
Leukopenia	56 (52.34)	51 (47.66)
Neutropenia	49 (45.79)	47 (43.93)
Vomiting	46 (42.99)	56 (52.34)
Abdominal pain	43 (40.19)	37 (34.58)
Pain	38 (35.51)	34 (31.78)
Dyspepsia	37 (34.58)	31 (28.97)
**The most common Grade 3, 4 AEs**		
Anaemia	15 (14.02)	10 (9.35)
Thrombocytopenia	13 (12.15)	14 (13.08)
Neutropenia	11 (10.28)	10 (9.35)
Diarrhoea	10 (9.35)	12 (11.21)
Alanine aminotransferase increased	3 (2.80)	5 (4.67)
Abdominal pain	2 (1.87)	1 (0.93)
Diarrhoea infectious	2 (1.87)	1 (0.93)
Pyrexia	2 (1.87)	1 (0.93)
Vomiting	0 (0.00)	3 (2.80)
**Serious AEs**		
	24 (22.43)	15 (14.02)

Data are presented as No (%) of patients

All of the AEs were assessed to define the causality relationship, and most of the AEs were possibly related to pertuzumab. No death was reported during the study.

Among 214 patients, the most common AE of grade 3 or 4 was anaemia (Table 5).

The number of patients who experienced a serious AE in the reference pertuzumab and P013 arms were 15 (14.02%) and 24 (22.43%), respectively. In the reference pertuzumab arm, 20 (90.91%) and in P013 arm 9 (75%) of the SAEs required in-patient hospitalization or prolongation of existing hospitalizations. Anaemia and thrombocytopenia were the most frequent SAEs in this study.

Four patients experienced significant decline in LVEF (≥10% points from baseline to < 50%) in the reference pertuzumab arm compared to two patients in the P013 arm (*P*-value = 0.68).

**Fig. 2:**
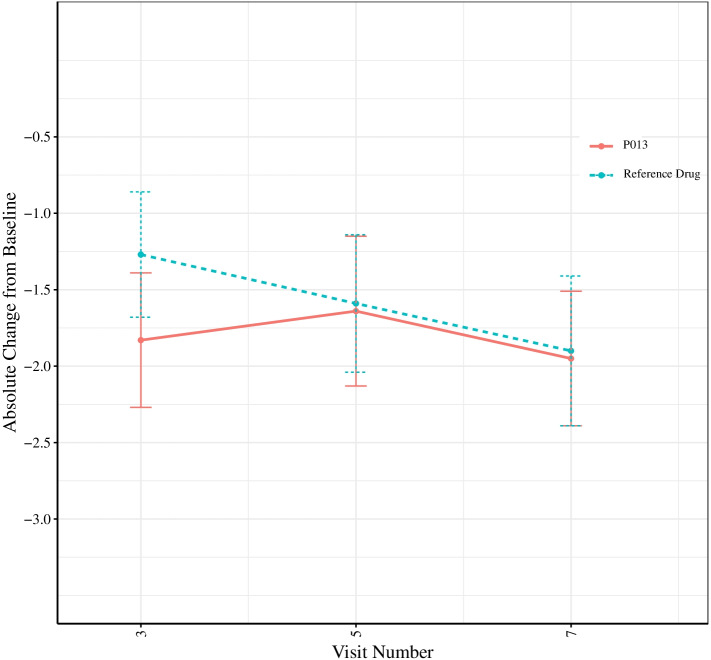
Absolute left ventricular ejection fraction (LVEF) change form baseline during treatment

Figure 2 illustrates the trend of the absolute change in LVEF in visits 3, 5, and 7 from baseline in the study population. This figure shows no significant decline in P013 arm compared to the reference drug.

#### Immunogenicity

All samples were negative for ADA in the two treatment groups. As there was no positive sample, statistical analysis could not be performed for the correlation between the ADA status and safety.

## Discussion

The results of this randomized, phase III trial demonstrated the equivalency of P013 with reference pertuzumab, when administered in the neoadjuvant setting in patients with HER2-positive breast cancer.

In our trial the bpCR rate in the control arm was considered to be 66% based on the TRYPHAENA trial [20]. The equivalency margin of 0.2 was defined according to the investigators’ opinion. Based on this margin, the minimum acceptable bpCR rate was considered to be 46% that is much higher than the range of 50–60% of the treatment effect (approximately 77%).

The study met its primary endpoint by showing equivalent bpCR among the two treatment groups. Secondary efficacy endpoints, including tpCR, ORR and BCS, were also comparable, and P013 was tolerable according to safety results.

In similar studies developing biosimilars, efficacy endpoints were similar to our study. Pivot et al. conducted a study comparing the efficacy, safety, and immunogenicity of SB3 (trastuzumab biosimilar) and reference trastuzumab in patients treated with neoadjuvant therapy for HER2–positive early breast cancer. The primary endpoint was bpCR and secondary endpoints were tpCR and ORR [21]. Some other studies investigating trastuzumab biosimilars, like Lammers et al., Stebbing et al., and von Minckwitz et al., had pCR as their primary endpoints and the latter had ORR, too [22–24]. Also, all these studies had safety and immunogenicity as their secondary outcomes [21–24]. Moreover, Lammers et al., Stebbing et al., and von Minckwitz et al. reported their results as pCR differences, like our study [22–24].

The benefits of dual anti-HER2 therapy has been noted in several studies. In a systematic review and meta-analysis done on ten randomized controlled trials, dual anti-HER2 therapy resulted in beneficial effects with respect to pCR rate (hazard ratio = 1.34, *p* = 0.0002) and was superior to single-agent antiHER2 therapy in patients with early breast cancer [25].

tpCR and pCR are both useful surrogate outcomes for assessing the efficacy of chemotherapeutic regimens in the neoadjuvant setting [8]. tpCR might be more informative. However, pCR is more widely in use as a primary outcome. Various studies have used this surrogate endpoint for assessing efficacy [13, 20]. Hence, as we aimed to compare our results with similar studies, we decided to use pCR as a primary surrogate outcome of efficacy.

The FDA approved pertuzumab for administration in neoadjuvant breast cancer treatment following the NeoSphere trial [13]. Patients with early HER2-positive breast cancer were randomized to treatment with pertuzumab in four treatment arms, with the primary endpoint of pCR rate in the breast. Among all the regimens administered, patients treated with pertuzumab, trastuzumab, and docetaxel demonstrated the highest rate of pCR. The addition of pertuzumab to THP regimen resulted in a pCR rate of 45.8% (49 of 107), showing a statistically significant improvement compared to THP (*p* = 0.0141). It is noteworthy that a 5-year follow up of this trial demonstrated that patients achieving tpCR (all groups combined) had longer progression-free survival (PFS) compared to patients without tpCR (hazard ratio = 0·54), with a rate of 86% in the docetaxel, carboplatin, and trastuzumab (TCH) group. Likewise, DFS results were highest in the TCH group (84%) [26].

Safety concerns associated with dual HER-2 blockade, particularly focused on cardiac safety, led to conducting the TRYPHAENA trial [20]. This randomized phase II trial on patients with early breast cancer also assessed pCR in the breast (ypT0/is). Pertuzumab combined with trastuzumab-based chemotherapy was assessed, and bpCR rate was consistently high and similar across all treatment groups (approximately 60%).

TCHP, as one of the treatment arms in this trial, had a promising effect on breast pCR. The PFS and DFS rates in this regimen were 87% (95% CI: 80–95) and 90% (95% CI: 82–97), respectively [27]. Therefore, this regimen was administered in our trial. In the TRYPHAENA trial, pCR rate of 66.2% was observed in the THCP arm, which is the closest to the pCR rate (67.62%) of the P013 group in our study.

The BERENICE trial conducted by Swain et al. evaluated neoadjuvant treatment regimens with pertuzumab, trastuzumab, and standard anthracycline- and taxane-based chemotherapy in patients with localized breast cancer. pCR in the breast and lymph nodes (tpCR) was approximately 60%, which is in line with our findings (56.05% in P013 and 63.55% in the reference pertuzumab arm) [28]. Similarly, tpCR rate in the TCHP arm of the phase III KRISTINE trial, was 56% [29]. Consistent with other studies, HR status influenced pCR rate in the treatment groups [13, 20, 29, 30]. HR-negative patients had higher bpCR rates in both arms (81.82% vs. 55.55% in P013 and 83.72% vs. 59.27% in reference drug arm).

Another secondary endpoint in our study was ORR. Similar to that reported in the NeoSphere trial, [13], the ORR was noted in 87.85 and 84.11% of the patients in P013 and reference pertuzumab groups, respectively. The ORR was 89.6% in the TCHP arm in the TRYPHAENA trial [20].

The BCS rate in our study was also comparable to other trials. 27% lumpectomy was reported following neoadjuvant TCHP systemic therapy [20]. In the subset of patients without inflammatory breast cancer in the KRISTINE trial, this regimen resulted in a 53% BCS rate [29]. However, controversies are raised about BCS accuracy for the assessment of treatment effectiveness. Several factors influenced the choice of surgery after systemic therapy. BCS was significantly less likely in tumors larger than 5 cm, multicentricity or multifocality of the tumors, or tumors with estrogen receptor–negative status [20]. In the subset of patients without inflammatory breast cancer in the KRISTINE trial, this regimen resulted in a 53% BCS rate [29]. Controversies are raised about BCS accuracy for the assessment of treatment effectiveness. Several factors influenced the choice of surgery after systemic therapy. BCS was significantly less likely in tumors larger than 5 cm, multicentric or multifocal tumors, or tumors with estrogen receptor–negative status [31, 32]. On the other hand, the two most essential factors in determining surgery type in the clinical setting are the surgeon’s recommendation and the patient’s personal preference. Even patients with pCR may undergo a mastectomy due to surgeon or patient’s decision, especially in developing countries compared to developed countries (*p* = 0.006) [33].

The overall safety profile of both TCHP arms in our study was consistent with the expected adverse reaction profile of the regimen and previous studies. In our study, aneamia, thrombocytopenia, and nausea were the most common AEs reported in both arms. In the TRYPHANEA trial, during the neoadjuvant period, diarrhea, alopecia and nausea (all grades) were reported in more than 50% of patients [20]. In the present study, aneamia, thrombocytopenia, and neutropenia were the most frequently reported grade 3–4 AEs in both arms. The high incidence of aneamia could be related to the docetaxel toxicity profile, as mentioned in the UpToDate for docetaxel, the incidence of aneamia is 65 to 97% [34].

The incidence of LVEF decrease in this study was also in accordance with previous studies, with four patients in the reference pertuzumab arm and two patients in P013 arm. In the BERENICE trial with the primary objective of evaluating cardiac safety of pertuzumab and trastuzumab in neoadjuvant chemotherapy, 13 patients (6.5%) and four patients (2.0%) in each arm experienced ≥1 LVEF decline [28].

Immunogenicity was negative in both treatment groups, indicating the low immunogenic potential of both drugs. The specificity of detected ADA for the therapeutic protein product is usually established by a confirmatory assay. Furthermore, ADAs are characterized by titration and neutralization assays. Since all samples were negative for ADA, no confirmatory and neutralizing assay were performed. This result is comparable with previously published data [28, 35].

## Conclusion

This Phase III, multicenter, randomized, triple-blind study demonstrated the equivalent efficacy and comparable tolerability of the biosimilar candidate P013 compared to the pertuzumab reference drug in women with locally advanced, inflammatory, or early HER2-positive breast.

## Supplementary Information

**Additional file 1: Supp. Table 1**. Breast cancer type. **Supp. Table 2**. Tumor and Lymph node distribution. **Supp. Table 3**. Frequency of cycles received in each group. **Sup. Figure**. Forest plot for bpCR result of PP and ITT analysis.

## Data Availability

The datasets generated and/or analysed during the current study are not publicly available due to the consent form but are available from the corresponding author on reasonable request.
