# Correction: Comparing efficacy and safety of P013, a proposed pertuzumab biosimilar, with the reference product in HER2-positive breast cancer patients: a randomized, phase III, equivalency clinical trial

**DOI:** 10.1186/s12885-022-10455-0

**Published:** 2022-12-22

**Authors:** Abolghasem Allahyari, Ali Ehsanpour, Behrouz Najafi, Nafseh Ansarinejad, Valiollah Mehrzad, Behjat Kalantari, Jahangir Raafat, Mojtaba Ghadiany, Farhad Shahi, Behrooz Gharib, Vahid Moazed, Adnan Khosravi, Mir Hossein Mirpour, Sina Salari, Seyedmohammadreza Mortazavizadeh, Amirabbas Nekoyi, Mohsen Khani, Alireza Sadeghi, Sirus Gharib, Alireza Bary, Mehrzad Mirzania, Shirin Haghighat, Seyed Mohsen Razavi, Seyed Amir Hossein Emami, Mehran Hosseinzadeh, Mahdi Mirbolouk, Sanambar Sadighi, Abdolali Shahrasbi, Ali Esfahani, Masoumeh Gity, Nassim Anjidani, Hamidreza Kaf, Safa Najaf

**Affiliations:** 1 Hematology Oncology Department, Faculty of Medicine, Mashhad University of Medical Sciences, Mashhad, Iran; 2 Thalassemia and Hemoglobinopathy Research Center, Health Research Institute, Ahvaz Jundishapur University of Medical Sciences, Ahvaz, Iran; 3 Hematology and Oncology Research Center, Razi Hospital, School of Medicine, Guilan University of Medical Science, Razi Hospital, Rasht, Iran; 4 Department of Hematology & Oncology, Iran University of Medical Sciences, Tehran, Iran; 5 Hematology and Oncology Department, Isfahan Medical School, Isfahan, Iran; 6 Hematology & Oncology, Department of Internal Medicine, School of Medicine, Shahid Bahonar Hospital, Kerman University of Medical Sciences, Kerman, Iran; 7 Mehrad Hospital, Tehran, Iran; 8 Department of Hematology and Medical Oncology, Shahid Beheshti University of Medical Sciences, Tehran, Iran; 9 Department of Hematology and Medical Oncology, Breast Disease Research Center, Cancer Institute, Imam Khomeini Hospital Complex, Tehran University of Medical Sciences, TUMS, Tehran, Iran; 10 Department of Medical Oncology & Hematology, Naft Hospital, Tehran, Iran; 11 Hematology & Oncology Kerman University of Medical Sciences, Kerman, Iran; 12 Hematology & Oncology, Department of Adult Hematology & Oncology, School of Medicine, Chronic Respiratory Diseases Research Center, National Research Institute of Tuberculosis and Lung Diseases, Dr. Masih Daneshvari Hospital, Shahid Beheshti University of Medical Sciences, Tehran, Iran; 13 Medical Oncology Guilan University of Medical Sciences, Razi Hospital, Fuman, Iran; 14 Medical Oncology, Hematology Taleghani Hospital, Shahid Beheshti University of Medical Sciences, Tehran, Iran; 15 Hematology/Oncology Yazd Azad University, Yazd, Iran; 16 Department of Hematology and Medical Oncology, Seyyed-al-shohada Hospital, Esfahan University of Medical Sciences, Tehran, Iran; 17 Medicine Saba Oncology Clinic, Isfahan, Iran; 18 Department of Hematology-Oncology, Isfahan University of Medical Sciences, Isfahan, Iran; 19 Hematology, Oncology Department of Internal Medicine, Guilan University of Medical Sciences, Guilan, Iran; 20 Department of Hematology & Oncology, Ghaem Hospital, Mashhad University of Medical Sciences, Mashhad, Iran; 21 Department of Hematology and Medical Oncology, Cancer Research Center, Cancer Institute of Iran, Imam Khomeini Hospital Complex, Tehran University of Medical Sciences, Tehran, Iran; 22 Hematology Research Center, Hematology and Medical Oncology Department, Shiraz University of Medical Sciences, Shiraz, Iran; 23 Iran Medical Science University, Tehran, Iran; 24 Medical Oncology, Cancer Institute, Imam Khomeini Hospital, School of Medicine, Tehran University of Medical Sciences, Tehran, Iran; 25 Hematology, Oncology Department of Internal Medicine, School of Medicine, Ahvaz Jundishapur University of Medical Sciences, Ahvaz, Iran; 26 Hazrat Rasol Hospital Rasht, Gilan, Iran; 27 Internal Medicine Group TUMS Faculty of Cancer Institute of Iran, Tehran, Iran; 28 Bouali Hospital, Tehran Medical Sciences, Islamic Azad University, Tehran, Iran; 29 Hematology and Oncology Research Center, Tabriz University of Medical Sciences, Tabriz, Iran; 30 Advanced Diagnostic and Interventional Radiology Research Center, Breast Disease Research Center, Imam Khomeini Hospital Complex, Tehran University of Medical Sciences, Tehran, Iran; 31 Medical Department, Orchid Pharmed Company, Tehran, Iran; 32 Breast Cancer Research Center, Motamed Cancer Institute, ACECR, Tehran, Iran

**Correction: BMC Cancer 22, 960 (2022)**

**https://doi.org/10.1186/s12885-022-09895-5**

Following publication of the original article [1], the authors identified that Behrouz Najafi was omitted as the third author in the author group. The author group in this correction article has been updated and the original article [1] has been corrected.
