# Supplementary material for: Comparing efficacy and safety of P013, a proposed pertuzumab biosimilar, with the reference product in HER2-positive breast cancer patients: a randomized, phase III, equivalency clinical trial
Source: BMC Cancer. 2022 Sep 7;22:960. doi: 10.1186/s12885-022-09895-5 (PMC9450379; doi:10.1186/s12885-022-09895-5)
Supplement: Supplementary file 1 — Additional file 1: Supp. Table 1. Breast cancer type. Supp. Table 2. Tumor and Lymph node distribution. Supp. Table 3. Frequency of cycles received in each group. Sup. Figure. Forest plot for bpCR result of PP and ITT analysis. [file 12885_2022_9895_MOESM1_ESM.docx]

*Supp. Table 1. Breast cancer type*

| **Tumor classification** | **Lymph Node classification** | **Anatomic Staging** | **Breast Cancer type** |
| --- | --- | --- | --- |
| T2 | N0 | IIA | Operable (Early Stage) |
| T2 | N1 | IIB | Operable (Early Stage) |
| T3 | N0 | IIB | Operable (Early Stage) |
| T2 | N2 | IIIA | Locally Advanced |
| T3 | N1 | IIIA | Operable (Early Stage) |
| T3 | N2 | IIIA | Locally Advanced |
| T4a | N0 | IIIB | Locally Advanced |
| T4b | N0 | IIIB | Locally Advanced |
| T4c | N0 | IIIB | Locally Advanced |
| T4d | N0 | IIIB | Inflammatory |
| T4a | N1 | IIIB | Locally Advanced |
| T4b | N1 | IIIB | Locally Advanced |
| T4c | N1 | IIIB | Locally Advanced |
| T4d | N1 | IIIB | Inflammatory |
| T4a | N2 | IIIB | Locally Advanced |
| T4b | N2 | IIIB | Locally Advanced |
| T4c | N2 | IIIB | Locally Advanced |
| T4d | N2 | IIIB | Inflammatory |
| T2 | N3 | IIIC | Locally Advanced |
| T3 | N3 | IIIC | Locally Advanced |
| T4a | N3 | IIIC | Locally Advanced |
| T4b | N3 | IIIC | Locally Advanced |
| T4c | N3 | IIIC | Locally Advanced |
| T4d | N3 | IIIC | Inflammatory |

*Supp. Table 2. Tumor and Lymph node distribution*

|  | Group | |
| --- | --- | --- |
| Tumor Classification | P013  N (%) | Pertuzumab  N (%) |
| T2 | 44 (41.12) | 46 (42.99) |
| T3 | 29 (27.19) | 22 (20.56) |
| T4a | 0 (0) | 1 (0.93) |
| T4b | 28 (26.17) | 28 (26.17) |
| T4c | 0 (0) | 1 (0.93) |
| T4d | 6 (5.61) | 6 (5.61) |
| Unavailable | 0 (0) | 3 (2.80) |
| Total | 107 (100) | 107 (100) |

|  | Group | |
| --- | --- | --- |
| Lym phnode Classification | P013  N (%) | Pertuzumab  N (%) |
| No | 16 (14.95) | 18 (16.82) |
| N1 | 55 (51.40) | 52 (48.60) |
| N2 | 14 (13.08) | 13 (12.15) |
| N3 | 22 (20.56) | 21 (19.63) |
| Unavailable | 0 (0) | 3 (2.80) |
| Total | 107 (100) | 107 (100) |

*Supp. Table 3. Frequency of cycles received in each group*

|  | Group | |
| --- | --- | --- |
| Frequency of cycles | P013  N | Pertuzumab |
| 1 | 0 (0) | 2 (1.87) |
| 2 | 1 (0.93) | 1 (0.93) |
| 4 | 0 (0) | 1 (0.93) |
| 5 | 3 (2.80) | 0 (0) |
| 6 | 103 (96.26) | 103 (96.26) |
| Total | 107 (100) | 107 (100) |


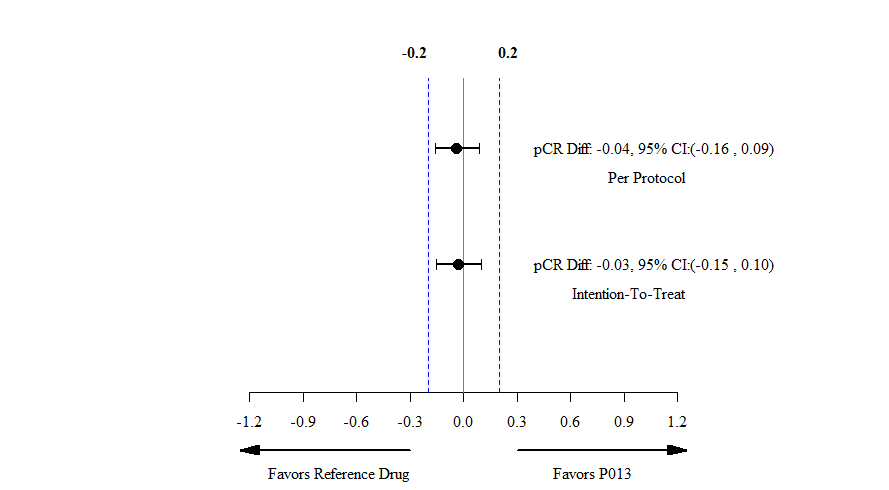


*Sup. Figure: Forest plot for bpCR result of PP and ITT analysis*
